# Update to CDC's Treatment Guidelines for Gonococcal Infection, 2020

**DOI:** 10.15585/mmwr.mm6950a6

**Published:** 2020-12-18

**Authors:** Sancta St. Cyr, Lindley Barbee, Kimberly A. Workowski, Laura H. Bachmann, Cau Pham, Karen Schlanger, Elizabeth Torrone, Hillard Weinstock, Ellen N. Kersh, Phoebe Thorpe

**Affiliations:** ^1^Division of STD Prevention, National Center for HIV/AIDS, Viral Hepatitis, STD, and TB Prevention, CDC; ^2^Department of Medicine, University of Washington, Seattle, Washington; ^3^Department of Medicine, Emory University, Atlanta, Georgia.

Sexually transmitted infections (STIs) caused by the bacteria *Neisseria gonorrhoeae* (gonococcal infections) have increased 63% since 2014 and are a cause of sequelae including pelvic inflammatory disease, ectopic pregnancy, and infertility and can facilitate transmission of human immunodeficiency virus (HIV) (*1*,*2*). Effective treatment can prevent complications and transmission, but *N. gonorrhoeae’s* ability to acquire antimicrobial resistance influences treatment recommendations and complicates control (*3*). In 2010, CDC recommended a single 250 mg intramuscular (IM) dose of ceftriaxone and a single 1 g oral dose of azithromycin for treatment of uncomplicated gonococcal infections of the cervix, urethra, and rectum as a strategy for preventing ceftriaxone resistance and treating possible coinfection with *Chlamydia trachomatis* (*4*). Increasing concern for antimicrobial stewardship and the potential impact of dual therapy on commensal organisms and concurrent pathogens (*3*), in conjunction with the continued low incidence of ceftriaxone resistance and the increased incidence of azithromycin resistance, has led to reevaluation of this recommendation. This report, which updates previous guidelines (*5*), recommends a single 500 mg IM dose of ceftriaxone for treatment of uncomplicated urogenital, anorectal, and pharyngeal gonorrhea. If chlamydial infection has not been excluded, concurrent treatment with doxycycline (100 mg orally twice a day for 7 days) is recommended. Continuing to monitor for emergence of ceftriaxone resistance through surveillance and health care providers’ reporting of treatment failures is essential to ensuring continued efficacy of recommended regimens.

Combination therapy, using a highly effective gonococcal therapeutic agent with cotreatment for chlamydia, has been recommended since 1985. In 2007, based on data from CDC's Gonococcal Isolate Surveillance Project* (GISP) indicating widely disseminated quinolone-resistant gonococcal strains in the United States, CDC no longer recommended fluoroquinolones for treatment, leaving cephalosporins as the only remaining recommended antimicrobial class (*6*). Availability of sensitive *C. trachomatis* nucleic acid amplification tests were widespread by 2010, but CDC recommended gonococcal dual therapy with a cephalosporin (ceftriaxone 250 mg IM or cefixime 400 mg orally) and either azithromycin or doxycycline (*4*) to reflect concerns regarding emerging gonococcal resistance. By 2011, the minimum inhibitory concentrations (MICs) of cefixime necessary to inhibit *N. gonorrhoeae* growth in vitro were increasing. In 2012, cefixime was no longer a recommended gonococcal regimen (*7*), with ceftriaxone and azithromycin combination therapy the only recommended regimen for uncomplicated gonorrhea (*5*). Since publication of the 2015 Sexually Transmitted Diseases (STD) Treatment Guidelines, concerns regarding antimicrobial stewardship have increased, especially the impact of antimicrobial use on the microbiome and data indicating azithromycin resistance (elevated MICs) for gonorrhea and other organisms (*1**,**3*). Pharmacokinetic and pharmacodynamic modeling has also affected the understanding of optimal antimicrobial dosing for *N. gonorrhoeae* treatment. This update provides the rationale for the change in gonorrhea treatment recommendations to a higher dose (500 mg) of ceftriaxone and removal of azithromycin from the recommended regimen.

During 2018, CDC staff members and subject matter experts identified essential questions regarding gonorrhea treatment to update the 2015 STD Treatment Guidelines (*5*). A literature search of PubMed, Embase, and Medline databases conducted for January 2013–May 2019 using the parameters (gonorrhea[MeSH]) OR (gonococcal[all fields] OR gonorrhea[all fields] OR “*Neisseria gonorrhoeae*”[all fields]) AND (treatment[MeSH] OR antibiotic[MeSH] OR therapy) generated >2,200 abstracts. Titles and abstracts were assessed, and 248 clinically relevant articles were reviewed. Abstracts from STD conferences held during 2015–2018 and on the National Institutes of Health clinical trials website (https://clinicaltrials.gov) were also reviewed.

GISP susceptibility data from January 2013 to May 2019 were reviewed. GISP monitors gonorrhea antimicrobial susceptibility patterns in the United States through monthly testing of urethral isolates from 25 symptomatic men in each of 25–30 STD specialty care clinics (*1*). Regional laboratories conduct antimicrobial susceptibility testing by agar dilution to determine MICs for selected antimicrobials. Although the Clinical and Laboratory Standards Institute (CLSI) has not established *N. gonorrhoeae* resistance breakpoints for ceftriaxone, cefixime, or azithromycin, CLSI categorizes isolates with MICs of ≤0.25 *μ*g/mL as susceptible for ceftriaxone and cefixime, and those with MICs of ≤1.00 *μ*g/mL as susceptible for azithromycin (*8*,*9*). To identify isolates with elevated MICs, GISP uses the following “alert values” to identify potential emerging resistance: MIC ≥0.125 *μ*g/mL for ceftriaxone, ≥0.25 *μ*g/mL for cefixime, and ≥2 *μ*g/mL for azithromycin (*1*).

In 2019, during an in-person meeting of governmental and nongovernmental participants, CDC staff members and subject matter experts reviewed data and presented their individual expert opinions. Each essential question was discussed, and applicable published articles were reviewed for their strengths, weaknesses, and relevance. Individual participants evaluated the quality of evidence, provided their input, and discussed findings in the context of the modified rating system used by the U.S. Preventive Services Task Force.^†^ CDC staff members independently reviewed tables of evidence,^§^ individual comments from the participants and professional organizations, and existing guidelines from other organizations to determine if revisions to the 2015 CDC STD Treatment Guidelines were warranted.

## Evidence and Rationale

**Antimicrobial stewardship**. The 2019 report on antimicrobial resistance threats in the United States (*3*) highlights that antimicrobial stewardship, i.e., the development, promotion, and implementation of activities to ensure the appropriate use of antimicrobials, remains a major public health concern. Data continue to document the impact of antimicrobials on the microbiome and on pathogenic organisms. A recent investigation comparing children who received twice-yearly azithromycin with children who received placebo found that the gut’s resistome, a reservoir of antimicrobial resistance genes in the body, had increased determinants of macrolide and nonmacrolide resistance, including beta-lactam antibiotics, among children receiving azithromycin (*10*). A higher proportion of macrolide resistance in nasopharyngeal *Streptococcus pneumoniae* was demonstrated in communities receiving mass administration of oral azithromycin (*11*). Azithromycin resistance has been demonstrated in another STI, *Mycoplasma genitalium*, and sexually transmissible enteric pathogens (e.g., *Shigella* and *Campylobacter*) (*12*–*14*). In addition, evidence supports increasing concern for the efficacy of azithromycin to treat chlamydial infections, especially rectal infections (*15*,*16*).

GISP data show that the ceftriaxone MIC50 and MIC90 (MIC required to inhibit growth of 50% and 90% of organisms, respectively) were only one doubling dilution higher during 2014–2018, compared with the respective ceftriaxone MIC50 and MIC90 during 1992–1995 (*1*). Although dual drug therapy with different mechanisms of action (ceftriaxone and azithromycin) might have mitigated emergence of reduced susceptibility to ceftriaxone in *N. gonorrhoeae*, concerns regarding potential harm to the microbiome and the effect on other pathogens diminishes the benefits of maintaining dual therapy as the recommended treatment regimen.

**Pharmacokinetic and pharmacodynamic considerations.** Ceftriaxone is a bactericidal third-generation cephalosporin with widely variable pharmacokinetics (*17*). Efficacy is best predicted by time that the serum free (i.e., unbound) drug concentration remains higher than the organism’s MIC (*f*T_>MIC_). Although no human data exist confirming the length of time above the MIC required to eradicate gonorrhea at different anatomic sites, using Monte Carlo modeling, ceftriaxone has been estimated to require concentrations higher than the strain MIC for approximately 20–24 hours for effective urogenital gonorrhea treatment (*18*). A 250 mg ceftriaxone dose does not reliably achieve levels higher than an MIC ≥0.125 *μ*g/mL for an extended duration (*18*). A murine model was used to estimate pharmacokinetic and pharmacodynamic parameters needed for cure at urogenital sites for both susceptible and resistant strains of *N. gonorrhoeae* (*19*). Investigators evaluated the efficacy of various ceftriaxone doses (0.06–30 mg/kg body weight). The lowest ceftriaxone dose that was 100% effective at eradicating the susceptible organism (MIC = 0.008 *μ*g/mL) 48 hours after treatment was 5 mg/kg body weight, which corresponded to an *f*T_>MIC_ of 23.6 hours, consistent with the Monte Carlo simulation (*18*,*19*). Translating into human doses, a 500-mg dose corresponds to 5 mg/kg body weight (80–100 kg) human, whereas 250 mg only corresponds to 3 mg/kg body weight for an 80 kg person.

The pharynx tends to be screened less often (*1*) than other anatomic sites, and globally, most reported ceftriaxone-based regimen treatment failures have involved treatment of pharyngeal gonorrhea (*20*). Ceftriaxone concentrations tend to be more variable in the pharynx, and treatment of *N. gonorrhoeae* likely requires longer times above the strain’s MIC (*21*,*22*). Continued uncertainty regarding ceftriaxone pharmacokinetics and pharmacodynamics in treating pharyngeal gonorrhea and the higher likelihood of treatment failures at this site strengthen the recommendation for an increase in the ceftriaxone dosage to 500 mg.

**Changes in azithromycin susceptibility.** Azithromycin resistance in *N. gonorrhoeae* is an increasing concern. Genomic epidemiology data confirm that azithromycin resistance can result from multiple mechanisms (*23*). Nationally, the percentage of *N. gonorrhoeae* isolates with reduced susceptibility (MIC ≥2.0 *μ*g/mL) increased more than sevenfold over 5 years (from 0.6% in 2013 to 4.6% in 2018) (Figure) (*1*). During 2018, among men who have sex with men, the proportion of GISP isolates with an azithromycin alert value was 8.6%, compared with 2.9% among men who have sex with women only (*1*). Studies have associated development of reduced azithromycin susceptibility with azithromycin exposure among patients with *N. gonorrhoeae* infection (*24*,*25*).

**FIGURE:**
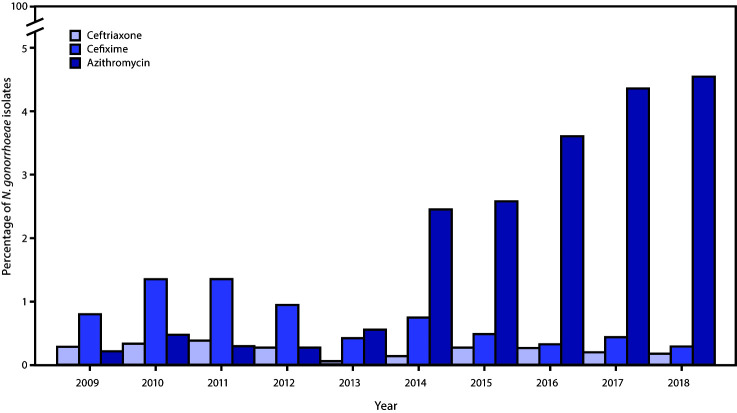
Percentage of *Neisseria gonorrhoeae* isolates with elevated minimum inhibitory concentrations (MICs)* to ceftriaxone, cefixime, and azithromycin — Gonococcal Isolate Surveillance Project, United States, 2009–2018 **Source:** CDC. Sexually Transmitted Disease Surveillance 2018. https://www.cdc.gov/std/stats18/default.htm. * Elevated MIC = ceftriaxone ≥0.125 *µ*g/mL; cefixime ≥0.25 *µ*g/mL; azithromycin ≥2.0 *µ*g/mL.

## Recommendations

For treatment of uncomplicated urogenital, rectal, or pharyngeal gonorrhea, CDC recommends a single 500 mg IM dose of ceftriaxone (Box). For persons weighing ≥150 kg (300 lbs), a single 1 g IM dose of ceftriaxone should be administered. If chlamydial infection has not been excluded, doxycycline 100 mg orally twice a day for 7 days is recommended. When ceftriaxone cannot be used for treating urogenital or rectal gonorrhea because of cephalosporin allergy, a single 240 mg IM dose of gentamicin plus a single 2 g oral dose of azithromycin is an option. Gastrointestinal symptoms, primarily vomiting within 1 hour of dosing, have been reported among 3%–4% of treated persons (*26*). If administration of IM ceftriaxone is not available, a single 800 mg oral dose of cefixime is an alternative regimen. However, cefixime does not provide as high, or as sustained, bactericidal blood levels as does ceftriaxone and demonstrates limited treatment efficacy for pharyngeal gonorrhea (*27*,*28*).

BOX **CDC recommended regimens for uncomplicated gonococcal infections, 2020**

**Regimen for uncomplicated gonococcal infections of the cervix, urethra, or rectum:**

Ceftriaxone 500 mg IM as a single dose for persons weighing <150 kg (300 lb)

- For persons weighing ≥150 kg (300 lb), 1 g of IM ceftriaxone should be administered.
- If chlamydial infection has not been excluded, providers should treat for chlamydia with doxycycline 100 mg orally twice daily for 7 days. During pregnancy, azithromycin 1 g as a single dose is recommended to treat chlamydia.

**Alternative regimens for uncomplicated gonococcal infections of the cervix, urethra, or rectum if ceftriaxone is not available:**

Gentamicin 240 mg IM as a single dose plus azithromycin 2 g orally as a single dose OR

Cefixime 800 mg orally as a single dose. If treating with cefixime, and chlamydial infection has not been excluded, providers should treat for chlamydia with doxycycline 100 mg orally twice daily for 7 days. During pregnancy, azithromycin 1 g as a single dose is recommended to treat chlamydia.

**Recommended regimen for uncomplicated gonococcal infections of the pharynx:**

Ceftriaxone 500 mg IM as a single dose for persons weighing <150 kg (300 lb)

- For persons weighing ≥150 kg (300 lb), 1 g of IM ceftriaxone should be administered.
- If chlamydia coinfection is identified when pharyngeal gonorrhea testing is performed, providers should treat for chlamydia with doxycycline 100 mg orally twice a day for 7 days. During pregnancy, azithromycin 1 g as a single dose is recommended to treat chlamydia.
- No reliable alternative treatments are available for pharyngeal gonorrhea. For persons with a history of a beta-lactam allergy, a thorough assessment of the reaction is recommended.*
- For persons with an anaphylactic or other severe reaction (e.g., Stevens Johnson syndrome) to ceftriaxone, consult an infectious disease specialist for an alternative treatment recommendation.

**Abbreviation:** IM = intramuscular.

* CDC. Sexually transmitted diseases treatment guidelines. MMWR Recomm Rep 2015;64(No. RR-3). https://www.cdc.gov/mmwr/preview/mmwrhtml/rr6403a1.htm.

In cases where gonococcal expedited partner therapy (provision of prescriptions or medications for the patient to take to a sex partner without the health care provider first examining the partner) is permissible by state law and the partner is unable or unlikely to seek timely treatment, the partner may be treated with a single 800 mg oral dose of cefixime, provided that concurrent chlamydial infection in the patient has been excluded. Otherwise, the partner may be treated with a single oral 800 mg cefixime dose plus oral doxycycline 100 mg twice daily for 7 days.

In cases of suspected cephalosporin treatment failure, clinicians should obtain relevant clinical specimens for culture and antimicrobial susceptibility testing, consult an infectious disease specialist or STD clinical expert (https://www.stdccn.org/) for guidance in clinical management, and report the case to CDC through state and local public health authorities within 24 hours. Health departments should prioritize notification and culture evaluation for the patient’s sex partner(s) from the preceding 60 days for those with suspected cephalosporin treatment failure or persons whose gonococcal isolates demonstrate reduced susceptibility to cephalosporins.

A test-of-cure is unnecessary for persons with uncomplicated urogenital or rectal gonorrhea who are treated with any of the recommended or alternative regimens; however, for persons with pharyngeal gonorrhea, a test-of-cure is recommended, using culture or nucleic acid amplification tests 7–14 days after initial treatment, regardless of the treatment regimen. Because reinfection within 12 months ranges from 7% to 12% among persons previously treated for gonorrhea (*29*,*30*), persons who have been treated for gonorrhea should be retested 3 months after treatment regardless of whether they believe their sex partners were treated. If retesting at 3 months is not possible, clinicians should retest within 12 months after initial treatment.

## Discussion

Continued support of gonorrhea prevention and control efforts remains fundamental, and preventing antibiotic resistance is crucial. The pharmacokinetics and pharmacodynamics of ceftriaxone indicate that a 500 mg dose in an average-weight U.S. adult achieves sufficiently high serum levels for an adequate duration to eradicate infection, even with wide pharmacokinetic variability. The high frequency of pharyngeal gonorrhea with substantial underscreening and the increased understanding of wide individual pharmacokinetic and pharmacodynamic variability has contributed to the recommendation for the increased ceftriaxone dose. These recommendations also include a test-of-cure for persons with pharyngeal gonorrhea to ensure eradication or detection of a possible treatment failure.

Emerging antimicrobial resistance affects gonorrhea treatment recommendations and other STIs. CDC recommends ceftriaxone monotherapy for treatment because *N. gonorrhoeae* remains highly susceptible to ceftriaxone, azithromycin resistance is increasing, and prudent use of antimicrobial agents supports limiting their use. Continuing to monitor for emergence of ceftriaxone resistance through surveillance and health care providers’ reporting of treatment failures will be essential to ensuring continued efficacy of recommended regimens.

**Summary**

**What is already known about this topic?**

*Neisseria gonorrhoeae* is an important cause of sexually transmitted infections that can have severe reproductive health consequences. *N. gonorrhoeae* can rapidly develop antibiotic resistance.

**What is added by this report?**

Based on review of recent evidence, CDC recommends a single 500 mg intramuscular dose of ceftriaxone for uncomplicated gonorrhea. Treatment for coinfection with *Chlamydia trachomatis* with oral doxycycline (100 mg twice daily for 7 days) should be administered when chlamydial infection has not been excluded.

**What are the implications for public health practice?**

Continuing to monitor for emergence of ceftriaxone resistance will be essential to ensuring continued efficacy of recommended regimens.

## Footnotes

* 2018 GISP sites: Albuquerque, New Mexico (1987–2018); Anchorage, Alaska (1987–2003, 2018); Atlanta, Georgia (1987–2018); Birmingham, Alabama (1987–2018); Boston, Massachusetts (1987–1992, 2014–2018); Buffalo, New York (2014–2018); Chicago, Illinois (1996–2018); Cleveland, Ohio (1991–2018); Columbus, Ohio (2012–2018); Dallas, Texas (1999–2018); Denver, Colorado (1987–2013, 2018); Greensboro, North Carolina (2002–2018); Honolulu, Hawaii (1987–2018); Indianapolis, Indiana (2013–2018); Jackson, Mississippi (2018); Kansas City, Missouri (1992–2001, 2007–2018); Las Vegas, Nevada (2002–2018); Los Angeles, California (2003–2018); Miami, Florida (1998–2013, 2018); Milwaukee, Wisconsin (2018); Minneapolis, Minnesota (1992–2018); New Orleans, Louisiana (1987–2018); New York, New York (2006–2018); Orange County, California (1991–2018); Phoenix, Arizona (1987–2018); Philadelphia, Pennsylvania (1987–2018); Pontiac, Michigan (2012–2018); Portland, Oregon (1987–2018); San Diego, California (1987–2018); San Francisco, California (1987–2018); Seattle, Washington (1987–2018); Tripler Army Medical Center, Hawaii (2001–2018); and Washington, DC (2018).

†
https://www.uspreventiveservicestaskforce.org/uspstf/grade-definitions .

§ https://www.cdc.gov/std/treatment-guidelines/evidence.htm.
